# Assessment of Xpert Bladder Cancer Monitor test performance for the detection of recurrence during non-muscle invasive bladder cancer follow-up

**DOI:** 10.1007/s00345-021-03629-1

**Published:** 2021-03-26

**Authors:** G. Cancel-Tassin, M. Roupret, U. Pinar, C. Gaffory, F. Vanie, V. Ondet, E. Compérat, Olivier Cussenot

**Affiliations:** 1 Sorbonne Université, GRC n°5, Predictive Onco-Urology, AP-HP, Hôpital Tenon, Service d’Urologie-Batiment Gabriel, 4 rue de la Chine, 75020 Paris, France; 2 CeRePP, Hôpital Tenon, 75020 Paris, France; 3 Sorbonne Université, GRC n°5, Predictive Onco-Urology, AP-HP, Hôpital Pitié-Salpêtrière, 75013 Paris, France

**Keywords:** Biomarkers, Cancer, Recurrence, Test, Urinary bladder neoplasm, Urothelial

## Abstract

**Purpose:**

To assess the performance of the Xpert Bladder Cancer (BC) Monitor during the follow-up of patients with non-muscle invasive bladder cancer (NMIBC).

**Methods:**

Patients with previously diagnosed NMIBC and followed up in clinical practice settings in two French urology departments between September 2017 and July 2019 were consecutively enrolled in this prospective observational study. Patients with a positive cystoscopy or computed tomography urogram underwent subsequent transurethral resection of the bladder, and/or biopsy, and the specimens were pathologically assessed. Cytology and Xpert BC Monitor tests were performed on urine samples. Xpert BC Monitor performance was assessed versus cystoscopy for disease-negative patients or versus histology for disease-positive patients, and was compared to that of cytology.

**Results:**

Overall, 500 patients with a median age of 70.0 years were included. NMIBC recurrence was diagnosed in 44 cases (8.8%). Overall sensitivity, specificity, and negative predictive values (NPVs) were 72.7% (32/44), 73.7% (330/448) and 96.5% (330/342) for the Xpert BC Monitor, and 7.7% (2/26), 97.8% (310/317) and 92.8% (310/334) for cytology, respectively. The Xpert BC Monitor detected 92.3% (12/13) of the high-grade tumours and ruled out their presence in 99.7% (330/331) of cases. Analysis of the areas under the receiver operating characteristic curves demonstrated the superior performance of the Xpert BC Monitor over that of cytology.

**Conclusion:**

Xpert BC Monitor performance was superior to that of cytology in the follow-up of NMIBC. The exclusion of aggressive tumours with a very high NPV (99.7%) supports the use of this urinary test in daily practice.

## Introduction

Approximately 75% of newly diagnosed bladder cancers are non-muscle invasive bladder cancers (NMIBCs) [1]. The 5-year recurrence (31–78%) and progression (0.8–45%) rates of NMIBC are high [2], requiring diligent and accurate follow-up for early detection as well as treatment of recurrence and/or progression. The follow-up schedule should be adapted according to the predicted European Organisation for Research and Treatment of Cancer (EORTC) risk score (i.e., low, intermediate or high) assigned at tumour diagnosis [2], and specific guidelines [3]. In most cases, white light cystoscopy and urine cytology are the gold standard for patient surveillance after NMIBC diagnosis [3]. The sensitivity of cytology is high for high-grade (HG) tumours, but low for low-grade (LG) tumours [4–6], and inter-observer reproducibility and intra-observer reproducibility are both poor [4]. White light cystoscopy also demonstrates a lack of sensitivity for flat lesions [7]. The procedure is unpleasant for patients, which can lead to non-compliance with follow-up schedules. In addition, surveillance of NMIBC patients with these methodologies is costly [8].

Using urine-based tumour markers in surveillance algorithms instead of cystoscopy and cytology could therefore reduce costs [9], and be quicker and easier to perform in clinical practice. Several urinary tests have been developed but, to our knowledge, none are currently being used in clinical practice. The Xpert Bladder Cancer (BC) Monitor (Cepheid, Sunnyvale, USA) is a qualitative in vitro diagnostic test that has been designed to monitor for the recurrence of bladder cancer. Using a voided urine specimen and the Cepheid GenXpert Instrument System to measure the expression of five mRNA targets frequently upregulated in patients with bladder cancer [10], this test has been validated in a prospective, multinational, multicentre study with data from 239 patients undergoing NMIBC surveillance [11]. The present study aimed to assess the performance of the Xpert BC Monitor in routine clinical practice during the follow-up of patients with NMIBC.

## Materials and methods

### Study design and setting

The TCC-GENE “Epidémiologie génétique et moléculaire des carcinomes urothéliaux” study was a French prospective observational study involving patients routinely followed up at the urology departments of the Tenon and La Pitié-Salpêtrière hospitals (AP-HP, Paris, France) between September 2017 and July 2019.

All patients with previously diagnosed NMIBC who were undergoing standard-of-care surveillance during the study period were eligible. Patients who underwent transurethral resection of the bladder (TURB) or *Bacillus* Calmette–Guérin treatment within the 6 weeks before enrolment were excluded.

### Study procedures

In both departments, white light cystoscopy was performed by experienced urologists. Patients with a positive cystoscopy or computed tomography (CT) urogram underwent subsequent TURB. Pathological examinations of the specimens, with samples assessed according to the WHO 2016 classification [12], were done by a referent uropathologist (EC).

The Xpert BC Monitor test measures the level of five target mRNAs (*ABL1*, *ANXA10*, *CRH*, *IGF2*, and *UPK1B*) by performing the real-time reverse transcriptase polymerase chain reaction (RT-PCR) in a self-contained cartridge. It uses the Cepheid GeneXpert Instrument System, which automates and integrates sample processing, nucleic acid amplification, and detection of the target sequences within approximately 90 min. A voided urine specimen (4.5 mL) from each patient was mixed with the Xpert urine transport reagent in the dedicated tube within one hour of urine collection. The test was then performed within seven days after transfer of 4 mL of the treated urine, using the pipette provided, to the sample chamber of the cartridge. Results were classified as “positive” or “negative” based on the proprietary linear regression algorithm built into the assay software, with “positive” results being defined as linear discriminant analysis (LDA) values of 0.5 or above. External controls were performed before the start of the study to confirm that results were in the expected range. Results from the Xpert BC Monitor were not used for patient management.

If sufficient amounts of the urine sample remained, cytology was performed according to the Paris system [13]. Cytology results were interpreted in a binary manner, with positive and suspicious results being considered as positive, and atypical and negative results being considered as negative.

### Blinding

To minimize bias in specimen analysis, both the operators performing Xpert BC Monitor testing and those performing cytology were blinded to patient status, cystoscopy, and histology results, as well as to the cytology and Xpert BC Monitor results, respectively. Urologists were also blinded to cytology and Xpert BC Monitor test results before cystoscopy.

### Sample size determination

White light cystoscopy and histology served as the reference methods for assessing the sensitivity of the Xpert BC Monitor (index test). In the clinical study by Valenberg et al. [11], the prevalence of positive patients was 15%, and the Xpert BC Monitor was 45% more sensitive than cytology. Assuming a 20% prevalence of positive patients based on our study centre data and an effect size of 25% when comparing the Xpert BC Monitor with cytology in this study, at least 320 patients were needed to detect a difference in sensitivity between the methods with a power of 80% and a two-sided *α* risk of 5%. The number of positive patients was evaluated after the recruitment of 300 patients to confirm that the assumed prevalence was correct and that the target for the number of positive cases had been met.

### Statistical analyses

Disease-positive cases were defined as those with a tumour of the bladder detected by cystoscopy and confirmed by pathological assessment. Xpert BC Monitor performance was assessed versus cystoscopy for disease-negative patients or versus histology for disease-positive patients, and was compared to that of urine cytology. For performance assessments, LG, HG, and carcinoma in situ (CIS) cases were categorically grouped as disease-positive cases. Invalid Xpert BC Monitor results and inconclusive cytology results were not considered; missing data were not replaced.

The sensitivity, specificity, positive predictive value (PPV), negative predictive value (NPV), receiver operating characteristic (ROC) curve, and area under the curve (AUC), together with the two-sided 95% confidence intervals (CIs), were determined for each test method. XLSTAT version 2020.2.2 (Addinsoft) was used to compare the AUC with a random test; this comparison was based on the difference between the AUC and 0.5 divided by the variance estimated using Bamber’s method. Success was achieved if the *p* value was less than 0.05.

## Results

**Fig. 1:**
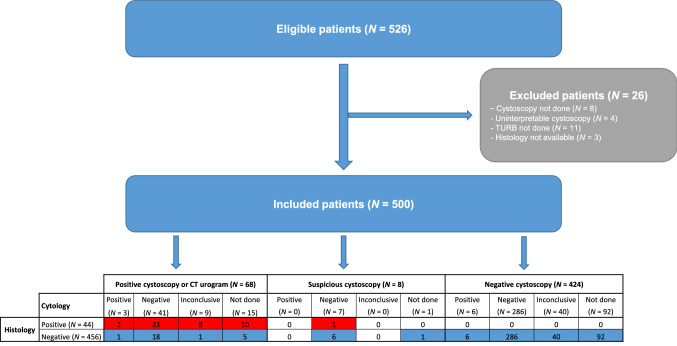
Study flow chart. *CT* computed tomography, *TURB* transurethral resection of the bladder

Of 526 patients enrolled in the study, 26 patients were excluded from the analyses (Fig. 1). The median age of the 500 included patients was 70.0 years (interquartile range 64.0–77.0), and the male-to-female ratio was 4:1 (Table 1). At diagnosis, 227 (45.4%) and 200 (40.0%) patients were at low and high risk, respectively, according to the EORTC classification. At the time of last resection, most tumours were stage Ta (72.0%) and histologically classified as LG (57.4%). Cystoscopy or CT urogram results were positive for 68 patients, and cystoscopy results were recorded as suspicious for eight patients (Fig. 1). Histology data confirmed NMIBC recurrence in 44 patients (8.8%). HG recurrence was identified in 13 patients (29.5%) and LG recurrence in 31 patients (70.5%).

**Table 1:** Patients’ demographic and tumour characteristics at enrolment (*N* = 500)

Characteristics	Result
Age (years), median (IQR)	70.0 (64.0–77.0)
Gender, *n* (%)	
Female Male	101 (20.2) 399 (79.8)
Smoking status, *n* (%)	
Current Former Never Unknown	82 (16.4) 177 (35.4) 126 (25.2) 115 (23.0)
Initial EORTC risk group, *n* (%)	
Low Intermediate High Unknown	227 (45.4) 39 (7.8) 200 (40.0) 34 (6.8)
Tumour stage at last resection, *n* (%)	
Ta T1 CIS^a^ Unknown	360 (72.0) 88 (17.6) 47 (9.4) 5 (1.0)
Tumour grade at last resection, *n* (%)	
Low High Unknown	287 (57.4) 194 (38.8) 19 (3.8)
Time since last resection (months), median (IQR)	21.0 (10.0–40.0)
EORTC highest risk group during surveillance, *n* (%)	
Low Intermediate High Unknown	175 (35.0) 78 (15.6) 236 (47.2) 11 (2.2)

*CIS* carcinoma in situ, *EORTC* European Organisation for Research and Treatment of Cancer, *IQR* interquartile range

^a^Including CIS alone, Ta + CIS, and T1 + CIS

Xpert BC Monitor tests on urine samples were valid in 98.4% of cases (*N* = 492/500). Cytology was performed on 392 urine samples and was inconclusive for 49 of them (12.3%) (Fig. 1). Xpert BC Monitor and cytology performance data are presented in Table 2. Overall sensitivity and specificity were 72.7% and 73.7% respectively for the Xpert BC Monitor, and 7.7% and 97.8% respectively for cytology. Overall NPVs were 96.5% for the Xpert BC Monitor and 92.8% for cytology. When LG recurrences were excluded, Xpert BC Monitor sensitivity increased to 92.3%, with 12 out of the 13 HG tumours being detected, and the NPV reached 99.7% (330/331). ROC curves showed that the diagnostic efficacy of the Xpert BC Monitor (AUC = 0.73, [95% CI 0.66–0.80], *p* < 0.0001) was higher than that for cytology (AUC = 0.53, [95% CI 0.48–0.58], *p* = 0.16), especially for the detection of HG tumours (AUC = 0.83, [95% CI 0.75–0.91], *p* < 0.0001 versus AUC = 0.55, [95% CI 0.44–0.65], *p* = 0.21). When considering the initial EORTC risk group, regardless of the tumour grade at recurrence, Xpert BC Monitor sensitivity was higher for patients with an intermediate (100.0%) or high (86.7%) risk at diagnosis than for those with a low risk at diagnosis (50.0%), and was greater than that for cytology in the three subgroups (Table 2). Xpert BC Monitor specificity was higher for patients with a low (82.3%) or intermediate (82.4%) risk at diagnosis than for those with a high risk at diagnosis (64.6%), whereas cytology specificity was very high in the three subgroups (97.2–100.0%). The Xpert BC Monitor NPV was very high in the three subgroups (94.4–100.0%). Analyses according to the highest risk group during NMIBC surveillance showed similar results (Table 2). No adverse events were recorded during the study.

**Table 2:** Performance of the Xpert Bladder Cancer (BC) Monitor and cytology, overall and according to recurrence grade, initial EORTC risk group, and the highest EORTC risk group during NMIBC surveillance

*n*/*N* (%) [95% CI]	Cytology	Xpert BC Monitor	Cytology	Xpert BC Monitor	Cytology	Xpert BC Monitor
	Overall		LG recurrences excluded		HG recurrences excluded	
Sensitivity	2/26 (7.7) [1.2; 25.5]	32/44 (72.7) [58.0; 83.7]	1/9 (11.1) [0.2; 46.0]	12/13 (92.3) [64.2; 100.0]	1/17 (5.9) [0.0; 29.3]	20/31 (64.5) [46.8; 78.9]
Specificity	310/317 (97.8) [95.4; 99.0]	330/448 (73.7) [69.4; 77.5]	310/317 (97.8) [95.4; 99.0]	330/448 (73.7) [69.4; 77.5]	310/317 (97.8) [95.4; 99.0]	330/448 (73.7) [69.4; 77.5]
PPV	2/9 (22.2) [0.0; 49.4]	32/150 (21.3) [14.8; 27.9]	1/8 (12.5) [0.0; 35.4]	12/130 (9.2) [4.3; 14.2]	1/8 (12.5) [0.0; 35.4]	20/138 (14.5) [8.6; 20.4]
NPV	310/334 (92.8) [90.0; 95.6]	330/342 (96.5) [94.5; 98.4]	310/318 (97.5) [95.8; 99.2]	330/331 (99.7) [99.1; 100.0]	310/326 (95.1) [92.7; 97.4]	330/341 (96.8) [94.9; 98.6]

	High initial EORTC risk		Intermediate initial EORTC risk		Low initial EORTC risk	
Sensitivity	1/9 (11.1) [0.2; 46.0]	13/15 (86.7) [60.6; 97.3]	0/4 (0.0) [0.0; 0.0]	5/5 (100.0) [100.0; 100.0]	1/11 (9.1) [0.0; 40.2]	10/20 (50.0) [30.0; 70.0]
Specificity	121/124 (97.6) [92.7; 99.5]	117/181 (64.6) [57.4; 71.2]	27/27 (100.0) [100.0; 100.0]	28/34 (82.4) [66.0; 91.9]	138/142 (97.2) [92.7; 99.1]	167/203 (82.3) [76.4; 86.9]
PPV	1/4 (25.0) [0.0; 67.4]	13/77 (16.9) [8.5; 25.3]	0/0 (0.0) [0.0; 0.0]	5/11 (45.5) [16.0; 74.9]	1/5 (20.0) [0.0; 55.1]	10/46 (21.7) [9.8; 33.7]
NPV	121/129 (93.8) [89.6; 98.0]	117/119 (98.3) [96.0; 100.0]	27/31 (87.1) [75.3; 98.9]	28/28 (100.0) [100.0; 100.0]	138/148 (93.2) [89.2; 97.3]	167/177 (94.4) [90.9; 97.8]

	Highest EORTC risk: high		Highest EORTC risk: intermediate		Highest EORTC risk: low	
Sensitivity	1/12 (8.3) [0.0; 37.9]	16/18 (88.9) [65.7; 97.9]	0/7 (0.0) [0.0; 0.0]	8/10 (80.0) [47.8; 95.1]	1/7 (14.3) [1.0; 53.6]	6/14 (42.9) [21.5; 67.4]
Specificity	149/152 (98.0) [94.0; 99.6]	137/214 (64.0) [57.4; 70.1]	53/53 (100.0) [100.0; 100.0]	55/68 (80.9) [69.8; 88.5]	100/104 (96.2) [90.1; 98.8]	131/157 (83.4) [76.8; 88.5]
PPV	1/4 (25.0) [0.0; 67.4]	16/93 (17.2) [9.5; 24.9]	0/0 (0.0) [0.0; 0.0]	8/21 (38.1) [17.3; 58.9]	1/5 (20.0) [0.0; 55.1]	6/32 (18.8) [5.2; 32.3]
NPV	149/160 (93.1) [89.2; 97.0]	137/139 (98.6) [96.6; 100.0]	53/60 (88.3) [80.2; 96.5]	55/57 (96.5) [91.7; 100.0]	100/106 (94.3) [89.9; 98.7]	131/139 (94.2) [90.4; 98.1]

*CI* confidence interval, *HG* high-grade, *LG* low-grade, *NPV* negative predictive value, *PPV* positive predictive value

## Discussion

Current European Association of Urology (EAU) guidelines on NMIBC [3] advocate repeated cystoscopies in all-risk patients as part of their 5-year follow-up after TURB: cystoscopies at 3 and 9 months, and then yearly for 5 years in low-risk patients; every 3 months for 2 years, then every 6 months until 5 years, and thereafter yearly in high-risk patients; and at an in-between (individualized) frequency in intermediate-risk patients. Urinary tests are used in the follow-up of NMIBC patients with the aim of reducing the frequency of such invasive testing, while still allowing the early detection of disease recurrence, the presence of recurrence to be excluded, and the detection of progression [14, 15]. In our prospective study, the Xpert BC Monitor test had a remarkably high NPV, excluding 96.5% of tumours overall and 99.7% of HG recurrences. Moreover, the sensitivity of the test was notably high for the detection of HG tumours (92.3%), with only one HG tumour going undetected. Our results also showed that the Xpert BC Monitor test performed well, regardless of whether the analysis was based on the initial EORTC risk group or the highest EORTC risk group during NMIBC surveillance. Therefore, either one of these assessments for risk could be used when deciding on the appropriate monitoring schedule using the Xpert BC Monitor test. Xpert BC Monitor testing was more accurate for patients with an intermediate or high risk of recurrence or progression, who had a higher cystoscopy frequency, than for low-risk patients. Our results therefore support the substitution of some cystoscopies by the Xpert BC Monitor test in NMIBC follow-up, allowing the frequency of the invasive cystoscopy procedure to be reduced without missing a recurrence that could have significant repercussions on bladder cancer progression [15].

Cytology and upper urinary tract imaging are also recommended in high-risk patients [3]. In our study, the Xpert BC Monitor success rate was higher than that for cytology, with only 1.6% of Xpert BC Monitor tests being invalid versus 12.3% of the cytology tests being inconclusive. The blind analysis of data from 500 consecutive patients showed that the Xpert BC Monitor had a higher sensitivity and a lower specificity than the cytology test, regardless of the recurrent tumour grade. Areas under the ROC curves demonstrated the superiority of the performance of the Xpert BC Monitor test over that of cytology for the detection of both all-grade and HG tumours. Our results are in line with those of other smaller studies (*N* = 140–432). Although the Xpert BC Monitor cannot replace cytology for the primary detection of tumours because of its lower specificity [16], it is a more objective tool than cytology. Indeed, the Xpert BC Monitor test is automated with a short hands‐on sample preparation time and single-use disposable cartridges; it should therefore give the same result wherever patients are managed, whereas cytology results are pathologist-dependent [4].

In their literature review on available urinary biomarkers for NMIBC surveillance, Soria et al. [15] concluded that the results of the study by Pichler et al. [6] were encouraging, but that other prospective trials were needed to validate the Xpert BC Monitor test. Since then, other studies investigating the accuracy of this test for NMIBC surveillance have been published [11, 17–19], which, together with the current study, have confirmed the results of Pichler et al. [6]. Using the Xpert BC Monitor urinary test between less frequent cystoscopies would therefore be more comfortable for patients and faster, while remaining safe. Moreover, in the context of the COVID-19 pandemic, the high NPV of the Xpert BC Monitor test might help to rule out patients with a negative result, avoiding unnecessary cystoscopies and the corresponding visits for patients at the appropriate level of risk. Concurrent telemedicine consultations with the urologist could help provide patients with a sense of security.

Limitations of our study include the small number of cytology tests analysed, limited due to a lack of available sample material. The percentage of recurrent tumours was also lower (8.8%) than that reported in other studies investigating Xpert BC Monitor accuracy in the follow-up of NMIBC (18.0–30.7%) [6, 11, 17, 18], but similar to that reported in a large-scale study investigating another urinary test (*N* = 127/1431, 8.9%) [20]. Nevertheless, patients were unselected in our prospective study, reflecting real-life clinical settings. Our study population, consisting of 500 analysed participants, was representative of patients with bladder cancer, mainly including men aged over 65 years, and current or former smokers [21, 22]. Moreover, our results confirm the findings of other studies [6, 11, 17, 18].

In conclusion, the very high NPV of the Xpert BC Monitor for the three initial or highest EORTC risk groups, and particularly for HG tumours, together with its high sensitivity for the detection of HG tumours, support the use of this urinary test in clinical practice to reduce the number of invasive cystoscopies in patients with NMIBC. Cost-effectiveness analyses are needed to further confirm the suitability of the widespread use of Xpert BC Monitor in clinical practice.
